# Free and total p-cresol sulfate levels and infectious hospitalizations in hemodialysis patients in CHOICE and HEMO

**DOI:** 10.1097/MD.0000000000005799

**Published:** 2017-02-10

**Authors:** Tanushree Banerjee, Timothy W. Meyer, Tariq Shafi, Thomas H. Hostetter, Michal Melamed, Yunnuo Zhu, Neil R. Powe

**Affiliations:** a Department of Medicine, University of California San Francisco, San Francisco; b Department of Medicine, Division of Nephrology, Veterans Administration Palo Alto Health Care System and Stanford University, Palo Alto, CA; c Department of Medicine, Division of Nephrology; d Welch Center for Prevention, Epidemiology and Clinical Research, Johns Hopkins University, Baltimore, MD; e Departments of Medicine and Epidemiology and Population Health, Albert Einstein College of Medicine, Bronx, NY; f Department of Medicine, Case Western University School of Medicine, Cleveland, OH; g Department of Medicine, Zuckerberg San Francisco General Hospital, San Francisco.

**Keywords:** hemodialysis, infection-related hospitalizations, p-cresol sulfate, uremic toxins

## Abstract

The uremic syndrome is attributed to progressive retention of compounds that, under normal conditions, are excreted by the healthy kidneys. p-cresol sulfate (PCS), a prototype protein-bound uremic retention solute, has been shown to exert toxic effects in vitro. Recent studies have identified relations between increased levels of PCS and indoxyl sulfate (IS) and adverse clinical outcomes in hemodialysis patients. We explored the relationship between free and total PCS and IS with infection-related hospitalizations (IH) and septicemia in 2 cohorts, Choices for Healthy Outcomes in Caring for end-stage renal disease (ESRD) Study (CHOICE) and Hemodialysis Study (HEMO).

We measured free and total levels of PCS and IS in stored specimens in CHOICE, a cohort of 464 incident hemodialysis patients enrolled in 1995 to 1998 and followed for an average of 3.4 years and in a prevalent dialysis cohort of 495 patients enrolled in HEMO from 1995 to 2000 and followed for an average of 4.4 years. We measured free PCS and IS using mass spectroscopy. The 2 cohorts were linked to United States Renal Data System (USRDS) Medicare billing records to ascertain IH over follow-up. We examined the association of free and total levels of PCS and IS with IH and septicemia using multilevel Poisson regression models adjusted for demographics, comorbidities, clinical factors, and laboratory tests including residual kidney function. We stratified patients a priori based on gastrointestinal (GI) disease as PCS and IS are produced in colon.

In CHOICE, highest tertile of free PCS in multivariable model was associated with 50% higher risk of IH [95% CI = 1.01–2.23] compared with lowest tertile in patients with no-GI disease. A significant trend was noted between greater levels of free PCS and septicemia in no-GI disease group in both cohorts, while no association was noted in GI disease group. Total PCS concentrations were not associated with either IH or septicemia in either cohort. No significant risk of IH or septicemia was noted with higher levels of free or total IS in either GI or no-GI disease group.

These results suggest an association between higher concentrations of free PCS and infection-related and sepsis-related hospitalizations in hemodialysis patients. Better methods of dialysis should be developed to evaluate the utility of removing PCS and its effect on the outcome and also therapies to decrease gastrointestinal tract production of uremic solutes.

## Abstract 2

Supplemental Digital Content is available in the text

## 1 Introduction

Hemodialysis patients are frequently accompanied by systemic infections. While there are many factors such as changes in gastrointestinal microbiota, poor nutritional status, malnutrition, vitamin D deficiency that predispose dialysis patients to infections,^[1]^ retention of uremic toxins may also contribute to immunodeficiency^[2]^ and increased susceptibility to infection.

In uremia, many protein-bound solutes such as p-cresol sulfate (PCS), indoxyl sulfate (IS), hippuric acid, N-phenylacetyl-glutamine, etc. accumulate.^[3]^ There has been interest in their contribution to uremic toxicity.^[4,5]^ PCS has been the most extensively studied of these solutes. Previous studies noted that PCS plasma levels rise out of proportion to urea that has toxic effects.^[6–8]^ Moreover, concentration of PCS, a main conjugate of p-cresol, has been linked to comorbidity and outcome parameters.^[9,10]^ Similar to protein-bound drugs, it can be expected that in most cases the free, nonprotein bound fraction will exert greater biological activity of protein-bound solutes.^[11,12]^ Since most of these uremic solutes compete with each other and with drugs for protein binding, the cumulative biologic impact of these multiple free solutes might substantially enhance the toxic effect of uremia.^[13,14]^ Recent literature has reported an association between free PCS concentrations and poor clinical outcomes in hemodialysis patients.^[15–18]^ Association between PCS and all-cause mortality has been variable including our own work showing no association with total PCS and all-cause mortality in Choices for Healthy Outcomes in Caring for end-stage renal disease (ESRD) Study (CHOICE)^[19,20]^ as well as in Hemodialysis Study (HEMO).^[21]^

We undertook this study to explore the relationship between protein-bound and free PCS and IS with infection-related hospitalizations (IHs) in 2 large repositories of serum samples from incident hemodialysis patients enrolled in CHOICE, a prospective observational study and from prevalent dialysis patients in the HEMO study, a randomized controlled trial of dialysis dose and flux.^[19,20]^ To our knowledge, very few dialysis studies^[15,16,18,19]^ have tested this relationship in patients with different comorbidities. These uremic retention solutes are generated in part in the gastrointestinal tract (GIT) and toxins generated in the intestine, such as advanced glycation end products, phenols, and indoles, may contribute to the pathogenesis of chronic kidney disease (CKD). It is thus biologically plausible, although not well recognized, that an important participant in the toxic load that contributes to CKD originates in the GIT. We therefore examined the relation in patients with the presence or absence of gastrointestinal (GI) disease to explore the role of GIT.

## 2 Subjects and methods

CHOICE was a national, prospective cohort study of incident hemodialysis and peritoneal dialysis patients. A total of 1041 dialysis patients were recruited from across the United States between October 1995 and June 1998 at a median of 45 days after the initiation of dialysis therapy (95% within 3.5 months). Study participants were enrolled from 81 dialysis clinics associated with Dialysis Clinic, Incorporated. Adult (ages 19–95 years), English or Spanish-speaking, and dialysis patients were eligible to participate in this study.

HEMO was a prospective, multicenter, randomized clinical trial of 1846 prevalent patients undergoing hemodialysis 3 times a week. The trial used a 2-by-2 factorial design to test the interventions of standard or high dose of dialysis, as measured by kt/V, and low-flux versus high-flux dialyzers. Randomization was performed centrally with the use of random permuted blocks, with stratification according to clinical center, age, and diabetes status. Patients were aged 18 to 80 years old, had been on hemodialysis for a minimum of 3 months, and were recruited between March 1995 and October 2000. In this cohort, patients were excluded if they were currently in an acute care of chronic care hospital, had an interdialytic 24-hour urine collection with a urea clearance >1.5 mL/min, pregnant, scheduled for a renal transplant within the period of the study, had active malignancies requiring chemotherapy or radiation therapy, had severe congestive heart failure, unstable angina pectoris, had symptomatic AIDS and active systemic infections, had chronic pulmonary disease, had cirrhosis with encephalopathy or abnormal PT, had severe malnutrition, used interventional drugs or involvement in other intervention protocols, unable to follow protocol due to mental incompetence, and unwilling to participate in the procedures of the protocol.^[22]^ For both cohorts, we selected participants with available samples.

All patients gave written informed consent before participation in the study.

### 2.1 Data collection

#### 2.1.1 Specimens

Blood was collected from CHOICE participants as part of routine quarterly lab draws. The detailed procedure has been described previously.^[19]^ Laboratory values for albumin, creatinine, hematocrit/hemoglobin, calcium, phosphate, and potassium, among others, were obtained from monthly lab draws at dialysis clinics. Blood was collected in HEMO at baseline and annually, appropriately handled and frozen at −80°. Study-related biochemical laboratory studies were done every 6 months locally. Routine laboratory variables available include serum albumin, creatinine, hematocrit, phosphate, and others.

#### 2.1.2 Uremic solutes

In CHOICE samples, total PCS and IS were assayed by high performance liquid chromatographic method with fluorescence detection at excitation 214 nm and emission 306 nm and free PCS and IS were assayed by LC/MS/MS with stable isotopic dilution plasma ultrafiltrate prepared using Nanosep 30K Omega separators (Pall, Ann Arbor, MI), as previously described.^[6,23]^ In HEMO samples, both total and free PCS and IS were assayed with the LC/MS/MS method. Our initial review of the data revealed some samples with very high values for free fractions of PCS and IS. Based on an external dataset of 119 freshly collected and processed plasma samples from 43 patients, we identified extreme values for these solutes. There is a possibility that these extreme values might represent problems with sample handling. We therefore excluded samples with extreme values of either PCS or IS from our analysis.^[19]^ For the purpose of this analysis, we defined extreme values as samples with either PCS or IS above 2 standard deviation of the mean based on external data [n = 87 (16.7%)]. For the remaining samples, we further excluded samples if either percent free PCS or IS values were >15% of total concentration (n = 40 [7.7%]).

#### 2.1.3 Other covariates

For CHOICE and HEMO^[22]^ participants, socio-demographic characteristics, marital status, type of insurance, and disability status were collected at baseline.

In CHOICE, cause of ESRD was taken from the Centers for Medicare and Medicaid Services (CMS) Medical Evidence report (CMS Form 2728). Medication use was defined as previously.^[24]^ In HEMO, a medical history form was administered at the enrollment visit.^[22]^

Comorbidity was assessed in CHOICE and HEMO using the index of coexistent disease, whose composite integer score ranges from 0 to 3 (with 3 as the highest severity level) and is a measure of both the presence and severity of comorbid conditions.^[25–28]^ Gastrointestinal disease included a history of or active esophagitis, gastritis, ulcers, pancreatitis, colitis, hiatal hernia, diabetic gastroparesis, reflux, diverticulosis, polyps, hemorrhage, or perforation. Access information was available in both cohorts; patients were characterized as having 1 of the 3 vascular access types: central venous catheter, arteriovenous fistula, or graft.

In CHOICE, dialysis dose (Kt/V) was calculated from clinic-supplied values of blood urea nitrogen, pre- and postdialysis weight, and dialysis duration using the Daugirdas formula.^[29]^ Residual kidney function (RKF) was obtained from the baseline self-report questionnaire and was defined as the ability to produce at least 250 mL of urine daily. In HEMO, routine urea kinetic modeling was performed monthly and extensive kinetics were performed at months 4 and 36.^[22]^ Timed urine collections for urea clearance were performed at baseline on all participants and annually for patients producing >50 mL of urine per day.^[22]^

#### 2.1.4 Outcomes

We used the United States Renal Data System (USRDS) hospitalization data (available through December 31, 2008) to determine the primary cause for each hospitalization during the study period. Our primary outcomes of interest were hospitalization due to: composite of all infections and prespecified cause of sepsis. We defined hospitalization using the primary *ICD-9* code 038.0–038.9 for septicemia or 790.7 for bacteremia.^[30]^ ICD coding information to determine hospitalization for sepsis has been used widely in other studies of sepsis.^[31,32]^ The sensitivity and specificity of *ICD-9* codes for sepsis have been evaluated by comparing *ICD-9* information with medical record review, using clinical consensus definitions of sepsis. The sensitivity of *ICD-9* codes was found to be 75.4% to 87.7%, depending on the specific *ICD-9* codes used.^[33]^ The positive predictive value of 038.x codes for sepsis was found to be 88.9% to 97.7%, depending on the clinical definition of sepsis.^[31]^ Only episodes in which the primary cause of hospitalization was sepsis were included in our analysis to avoid including cases in which infection was acquired during hospitalization.

IH was a predefined secondary outcome of the HEMO study. All hospitalizations of study participants were reported by the clinical center to the coordinating center and were adjudicated.^[34]^

#### 2.1.5 Statistical analysis

We compared patient characteristics by tertiles of PCS using *χ*^2^ tests for categorical variables and ANOVA for continuous variables. We used the Kolmogorov–Smirnov test to check normality. The Kruskal–Wallis test was used for the continuous variables if the normality assumption of the residuals was not met. We used the Fisher exact test for categorical variables when the expected cell frequency was less than 5.

Covariates with missing values in CHOICE included body mass index (5.6%), RKF (3.7%), phosphate (9.4%), creatinine (9.2%), albumin (9.4%), and Kt/VUREA (22.7%), while in HEMO the covariates with missing values were albumin (11.8%), phosphate (10.5%), and creatinine (10.3%). To avoid list-wise deletion,^[35]^ we imputed missing data with 15 data replicates using multiple imputations by the chained equations method implemented by the ice program in STATA. We used multivariable Poisson regression models to study the association between PCS levels, both total and free concentrations, and risk of infectious hospitalizations and septicemia. Potential confounders included in the model were age at enrollment, gender, race/ethnicity, index of coexistent disease score, albumin, creatinine, phosphate levels. Multicollinearity of the predictor variables was assessed using variance inflation factors. We incorporated methods to account for confounding by clinic site in CHOICE.^[36]^ To explore the role of GI disease, we stratified our model by the presence or absence of GI disease as PCS is generated by gut microbiota and the ensuing micro-biometabolome plays a significant role in proliferation of uremic retention solutes.

Sensitivity analyses included analyses of the full cohort without excluding extreme values. We further examined the association of free and total levels of IS with infection-related and sepsis hospitalizations in both CHOICE and HEMO. Statistical analyses were performed using STATA software, version 12.1 (Stata Corp. www.stata.com) and SAS 9.3. Statistical significance was defined as *P* < 0.05 using 2-tailed tests.

### 2.2 Institutional Review Board approval

This study was approved by the University of California, San Francisco, Institutional Review Board (Committee on Human Research application #10-00758).

## 3 Results

### 3.1 Baseline characteristics and PCS levels

There were 521 hemodialysis participants with available stored samples. Of these, we included 394 patients in this prospective study after excluding very high values for the free fractions of PCS and IS as described in the Methods (n = 127). Participants who were included in the study were less likely to be white or have cardiovascular disease (CVD), had slightly lower urea, hemoglobin, and higher albumin as compared with those excluded. We studied the association of free and protein-bound concentrations of PCS with IHs in these remaining 394 patients. Of these study participants (mean age: 57.2 years; 54.8% males and 45.2% females), 69 (17.5%) had hypertension, 69 (17.5%) had diabetes, 41.1% had GI disease (Table 1).

**Table 1:**
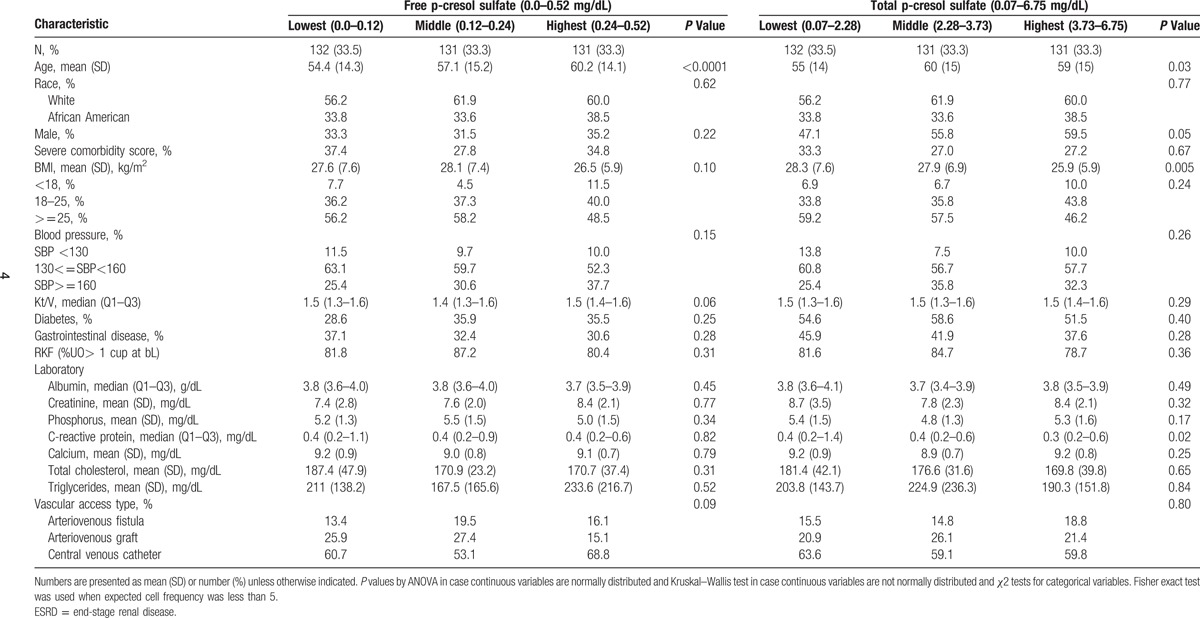
Selected characteristics of study participants by levels of free and total p-cresol sulfate in the choices for healthy outcomes in caring for ESRD study (N = 394).

We studied 485 patients in HEMO who had complete information on hospitalizations. Of these patients, 138 patients had very high values of free and total levels of PCS and IS and were excluded. Participants who were included compared with those excluded from the study were more likely to be black, had lower likelihood of CVD, and had slightly higher urea and urine volume. The mean age of these patients was 57.5 years, of whom 43.2% were males, 45.1% had diabetes, and 37.4% had GI disease (Table 2).

**Table 2:**
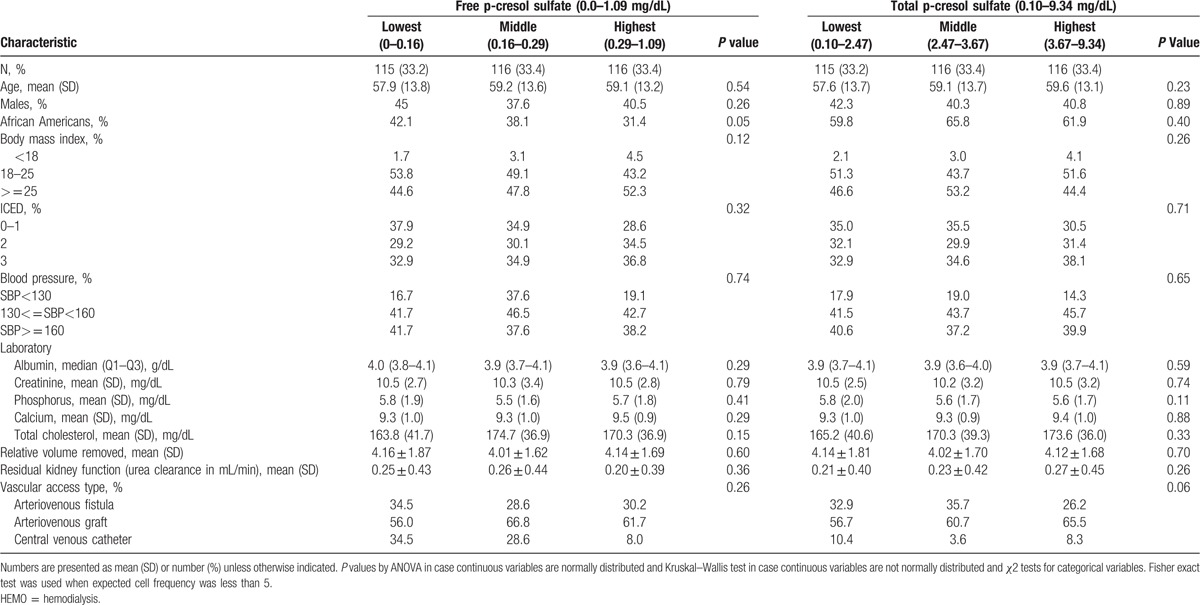
Selected characteristics of study participants by levels free and total p-cresol sulfate in the HEMO study (N = 347).

### 3.2 Association between free PCS fractions and IH

In CHOICE, test for interaction involving GI disease status with PCS fractions was significant (*P* = 0.03), demonstrating that the increased likelihood of risk of IH in patients with higher fractions of PCS was modified by GI disease. In univariate analysis, only cases with higher fractions of PCS had a higher risk of IH in patients with no-GI disease. Hospitalization risks of patients with moderate fractions of PCS compared with the lower fractions were consistently and markedly lower. A multivariable model, adjusted for demographics, comorbidity score, obesity, diabetes, CVD, RKF, albumin, creatinine, and phosphate, changed risk-ratio estimates only slightly (Table 3). The risk of hospitalization in the highest tertile of free PCS for patients without GI disease was 50% higher than the lowest tertile of free PCS. In the middle tertile of free PCS, the risk of hospitalization was not significant compared with the lowest tertile (RR [95% CI]: 0.91 [0.61–1.34]). A significant trend was observed between the higher concentrations of PCS with IHs in patients with no-GI disease (Ptrend = 0.01). In patients with GI disease, there were no significant associations observed between the fractions of PCS and risk of hospitalizations in the multivariable model (Table 3).

**Table 3:**
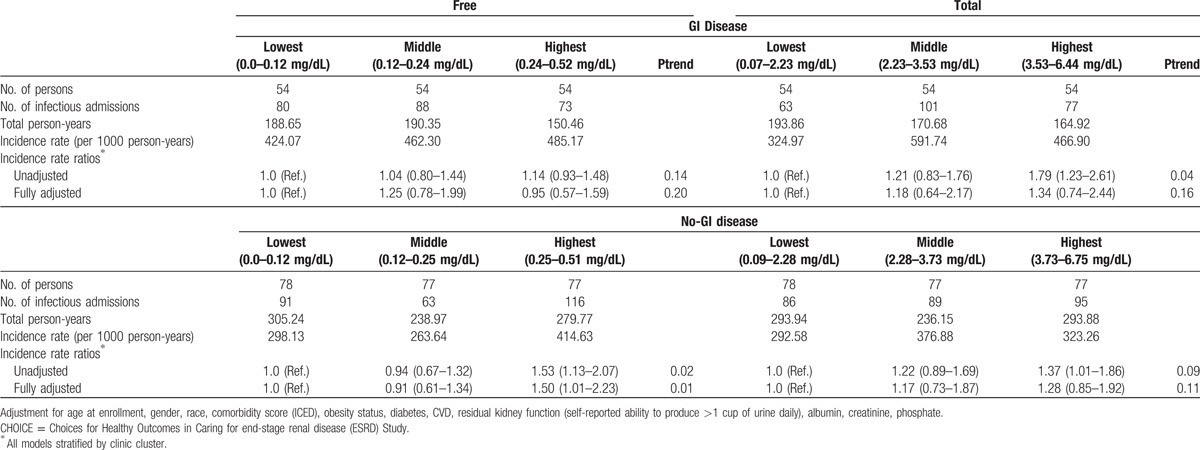
Crude estimates and incidence rate ratios of all infectious hospitalizations for free and total p-cresol sulfate stratified by gastrointestinal (GI) disease in CHOICE.

Supplementary Table 1 (ST 1) presents the association of PCS with IH in HEMO. We noted a borderline significant interaction between fractions of PCS and GI disease in HEMO (p-interaction = 0.05). We observed a significant trend across the tertiles of PCS in the multivariable model (p-trend = 0.02). The risk of hospitalization due to infection associated with the tertiles of PCS in patients with no-GI and GI diseases was not significant. Moreover, we did not observe any significant trend in higher concentrations of PCS with IHs as well.

### 3.3 Association between total PCS levels and IH

The results were similar to what obtained with free fractions of PCS in CHOICE. We found the test of interaction between GI disease status and PCS had a borderline *P* value (0.045). In multivariable model, the risk of infectious hospitalization associated with highest and middle tertile of PCS in the no-GI disease group was 28% and 17% higher than the lowest tertile, but this association was not significant. We again did not see any significant associations between higher levels of PCS with IHs in the GI disease group (RH [95% CI]: 1.18 [0.64–2.17] for middle tertile and 1.34 [0.74–2.44] for highest tertile) (Table 3).

Analyzing the association between levels of PCS and IHs in HEMO, we found a lower risk of IHs with greater levels of PCS, but this association was not significant. The association between greater levels of PCS and IHs in the GI disease group was in the same direction as in the no-GI disease group. Again, the associations were not significant (ST 1).

### 3.4 Association between free PCS fractions and sepsis hospitalizations

In univariate analysis in CHOICE, the risk of sepsis admission in the highest tertile of PCS was 68% higher compared with the lowest tertile in the no-GI disease group (95% CI: 0.87–3.24). The strength of the association of PCS with sepsis admissions though was attenuated after adjustment for confounders but the association remained nonsignificant. Among the patients with no-GI disease, there was a significant trend of an association of greater PCS fractions with risk of sepsis hospitalizations (Ptrend = 0.04) (Table 4). On the other hand, in patients with GI disease, the risk associated with sepsis admissions was 2.3-fold higher in the middle tertile and 1.3-fold higher in the highest tertile of PCS when compared with the lowest tertile. Furthermore, we observed no significant trend between the association of higher fractions of PCS with sepsis hospitalizations (Ptrend > 0.05) (Table 4).

**Table 4:**
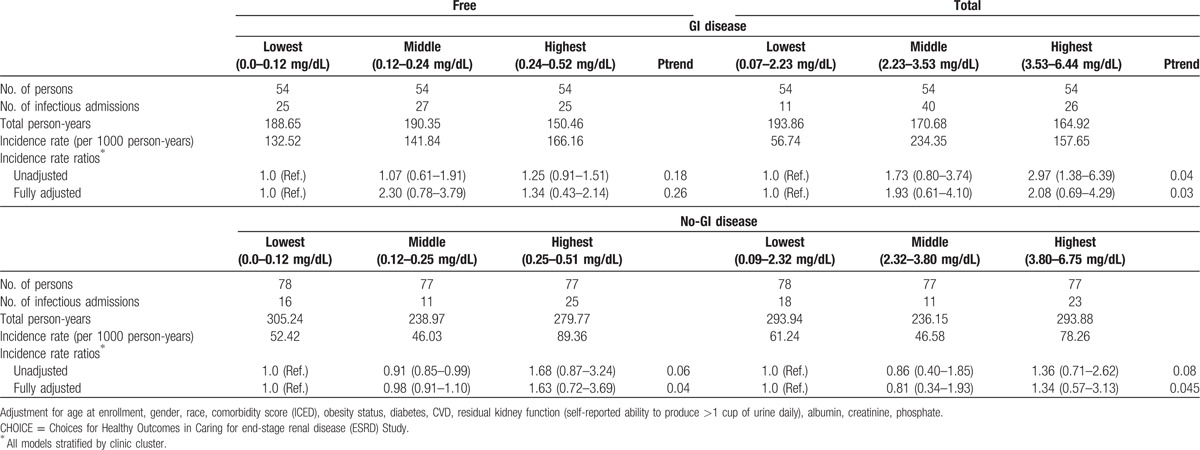
Crude estimates and incidence rate ratios of all sepsis hospitalizations for free and total p-cresol sulfate stratified by gastrointestinal (GI) disease in CHOICE.

The effect of PCS fractions on risk of sepsis hospitalization was again analyzed in the GI and no-GI disease groups in HEMO. In the no-GI disease group, there was no statistically significant difference in sepsis admissions observed in PCS highest tertile compared with the lowest tertile. In contrast, free fractions of PCS seemed to have a significant trend to correlate with sepsis-admissions (Ptrend = 0.03). The GI disease group also did not exhibit any significant association between PCS and sepsis admissions across the tertiles. A nonsignificant trend toward risk of sepsis admissions for PCS fractions was observed (Ptrend = 0.12 (ST 2)).

### 3.5 Association between total PCS levels and sepsis hospitalizations

The results were similar to our findings for free PCS fractions in the no-GI disease in CHOICE. Again, we observed greater risk of sepsis hospitalizations with higher levels of PCS, but the associations were not significant. However, a trend was observed which was borderline significant (Ptrend = 0.045) (Table 4). In GI disease, our results were in contrast to what we obtained with free PCS fractions. Here, we observed a graded risk of sepsis hospitalizations with increased levels of PCS (Ptrend = 0.03) (Table 4).

In HEMO, we did not observe a significant risk of sepsis hospitalizations with higher levels of PCS either in no-GI or GI disease group. Further, no significant trend was observed for risk of sepsis hospitalizations with increasing levels of PCS either in the 2 groups (Ptrend > 0.05) (ST 2).

### 3.6 Sensitivity analyses

#### 3.6.1 All participants

On analyzing the full cohort without excluding extreme observations, that is n = 521 in CHOICE and n = 485 in HEMO, showed results similar to the primary analysis (Supplementary Table ST3-ST6).

#### 3.6.2 Association of IS with infection-related and sepsis hospitalizations

Baseline characteristics of participants across the tertiles of IS in CHOICE and HEMO are presented in Supplementary tables ST7–ST8. In both CHOICE and HEMO, we did not observe a significant risk of infection-related or sepsis hospitalizations with higher levels of free IS in either GI or no-GI disease group. Our findings were again not significant when the association was examined with the total levels of IS (Supplementary Table ST9–ST12).

## 4 Discussion

This study was designed to evaluate the influence of protein bound and free concentrations of the uremic retention solute p-cresol and indoxyl sulfate on risk of hospitalization for infection in 2 national cohorts. We studied our hypothesis in both an incident and a prevalent dialysis cohort. Testing our hypothesis in both cohorts adds to the strength of replication of findings. We excluded patients from both cohorts with very high values of free and protein-bound concentrations of PCS and IS. For sepsis hospitalizations, a significant trend was noted with greater concentrations of free PCS in patients with no-GI disease in both CHOICE and HEMO. There was no significant relationship observed between the total concentrations of p-cresol with sepsis hospitalizations either in CHOICE or HEMO although we observed a significant trend between increasing levels of total PCS and sepsis admissions in CHOICE irrespective of GI disease status. No significant risk of infection-related or sepsis hospitalizations was noted with higher levels of free or total IS in either GI or no-GI disease group.

PCS is the main metabolite of p-cresol in humans,^[6,37]^ and it has a pro-inflammatory effect of leucocytes in CKD patients.^[23]^ Further evidence has indicated that p-cresol plays a major role in endothelial dysfunction, a characteristic of uremic syndrome.^[38]^ As a consequence, p-cresol retention has been considered as playing a role in the susceptibility of the uremic patients to infection.^[39]^ It is still unclear whether the total or free fraction of protein-bound uremic solutes is responsible for their toxic effect.^[40]^ It is reasonable to suppose that p-cresol along with indoxyl sulfate is linked to episodes of infection in dialysis patients. Our model found that after adjustment for other confounders, only free serum fractions of p-cresol in patients with absence of GI disease conferred significant risk to IHs. The observed effect of free p-cresol was prominent in hospitalizations with any form of infection and not limited to a specific infection-related hospitalization such as sepsis admission. Our findings corroborate with previous literature that found serum-free p-cresol was closely associated with IH in hemodialysis patients after 20-month follow-up.^[16]^ Despite the similarity in results, these 2 studies differed in their methodology. In the study by Lin et al, 100 stable hemodialysis patients were recruited from a single medical center and followed up till 20 months. During follow-up, events such as infection events, cardiovascular events, and cause of death were reviewed by 1 independent physician who was blinded for the study. Lin et al included only 1 episode of the event per subject for analysis. On the other hand, in both CHOICE and HEMO, patients were recruited from multiple dialysis clinics. For analysis purposes, we considered the count of infection-related hospitalization per patient.

### 4.1 Role of gastrointestinal disease

In our study, the risk of hospitalization associated with p-cresol levels in the absence of GI disease could be expected by the juxtaposition of p-cresol concentration and gut microbiome. The awareness that risk of hospitalizations associated with p-cresol concentration is related to the intestinal status stemmed in part from the insight that several toxins active in the uremic syndrome originate from the intestine. The microbiota and its metabolic activities such as proteolytic fermentation influenced by diet and transit time impact the production of PCS. On the other hand, we did not find any significant risk of hospitalization for infection due to increased concentration of p-cresol in patients with GI disease. We would expect that in the presence of GI disease the gut microbiome is affected and thus results in lower production of uremic solutes. However, some studies have suggested that uremic solutes once produced in gastrointestinal tract may promote inflammation and impair the intestinal barrier in patients with ESRD.^[41,42]^ This may lead to the spread of substances that could augment the impact of a uremic toxin on the kidneys. Such events can contribute to higher risk of comorbidities in dialysis patients. Although there was no significant association between serum concentrations of PCS and IH in the fully adjusted models in patients with GI disease, significant risk with higher levels of protein-bound p-cresol in CHOICE cohort was noted when the model was adjusted for demographics. Our finding corroborated the findings of previous studies^[43,44]^ that the gut microbiome is a link between uremic syndrome and comorbidity.

These findings might shed light on the present view of pathophysiologic events in uremia. We found an important relationship between free fraction of p-cresol and hospitalization in incident dialysis patients. Traditionally, several factors have been suggested to play a role in the induction of hospitalization for infection, such as diabetes because of impaired immunologic defense mechanisms and deficient phagocytic function, age as immune system declines with age, low serum albumin, and dialyzer membrane bioincompatibility.^[45–47]^ On the basis of the present data, the liberation of protein-bound solutes from their binding sites, which enhances their toxicity, should be considered an additional mechanism.

Our study has limitations. First, we measured solutes at a single time point and levels of these solutes likely vary over time. Repeated solute measurements might reveal a stronger association of solute levels with outcome. Second, our 2-cohort analysis may establish association between the uremic solute levels and risk of hospitalizations but does not infer causality.

In conclusion, our data indicates that accumulation of the uremic solute p-cresol in free form is associated with increased admissions of hospitalizations due to infections. This effect is consistent with a clinical study showing a link between high concentrations of plasma-free p-cresol and hospitalization rates for infectious diseases.^[13]^ These findings highlight the importance of developing better dialysis techniques to control uremic toxicity, while reducing harm and inconvenience to the patient and also therapies to decrease gastrointestinal tract production of PCS and IS. Future studies studying the interaction between the gut microbiome and kidneys are of paramount importance. Understanding this mechanism might result in developing novel targeted therapeutic interventions.

## Acknowledgments

The authors thank the patients, staff, laboratory, and physicians of Dialysis Clinic Inc (DCI), New Haven CAPD, and St. Raphael's Hospital for their participation in the CHOICE Study.

## Supplementary Material

Supplemental Digital Content
