# Supplementary material for: Free and total p-cresol sulfate levels and infectious hospitalizations in hemodialysis patients in CHOICE and HEMO
Source: Medicine (Baltimore). 2017 Feb 10;96(6):e5799. doi: 10.1097/MD.0000000000005799 (PMC5312983; doi:10.1097/MD.0000000000005799)
Supplement: Supplemental Digital Content [file medi-96-e5799-s001.doc]

Table ST1: Crude estimates and incidence rate ratios of all **infectious hospitalizations** for free and total P-cresol sulfate stratified by GI disease in HEMO

|  | **Free** | | | | **Total** | | | |
| --- | --- | --- | --- | --- | --- | --- | --- | --- |
|  | **GI Disease** | | | | | | | |
|  | **Lowest**  **(0.00-0.16 mg/dl)** | **Middle**  **(0.16-0.29 mg/dl)** | **Highest**  **(0.29-1.09 mg/dl)** | **PTrend** | **Lowest**  **(0.10-2.47 mg/dl)** | **Middle**  **(2.47-3.67 mg/dl)** | **Highest**  **(3.67-6.33 mg/dl)** | **PTrend** |
| No. of persons | 49 | 46 | 47 |  | 48 | 47 | 47 |  |
| No. of infectious admissions | 100 | 86 | 86 |  | 95 | 89 | 88 |  |
| Total person-years | 231.90 | 215.01 | 216.46 |  | 228.23 | 219.61 | 215.53 |  |
| Incidence rate (per 1000 person-years) | 431.22 | 399.97 | 397.30 |  | 416.25 | 405.26 | 408.29 |  |
| Incidence rate ratios* |  |  |  |  |  |  |  |  |
| Unadjusted | 1.0 (Ref.) | 0.88 (0.70-1.11) | 1.19 (0.95-1.48) | 0.12 | 1.0 (Ref.) | 1.23 (0.96-1.57) | 1.08 (0.83-1.39) | 0.56 |
| Fully- Adjusted | 1.0 (Ref.) | 0.88 (0.70-1.13) | 1.18 (0.94-1.48) | 0.13 | 1.0 (Ref.) | 1.11 (0.82-1.51) | 1.06 (0.79-1.46) | 0.66 |
|  |  |  |  |  |  |  |  |  |
|  | **No-GI Disease** | | | | | | | |
|  | **Lowest**  **(0.01-0.17 mg/dl)** | **Middle**  **(0.17-0.28 mg/dl)** | **Highest**  **(0.28-1.02 mg/dl)** |  | **Lowest**  **(0.11-2.46 mg/dl)** | **Middle**  **(2.46-3.66 mg/dl)** | **Highest**  **(3.66-9.34 mg/dl)** |  |
| No. of persons | 69 | 68 | 68 |  | 69 | 68 | 68 |  |
| No. of infectious admissions | 150 | 133 | 126 |  | 146 | 137 | 126 |  |
| Total person-years | 344.28 | 328.61 | 333.36 |  | 347.65 | 334.05 | 324.56 |  |
| Incidence rate (per 1000 person-years) | 435.69 | 404.73 | 377.97 |  | 419.96 | 410.11 | 388.22 |  |
| Incidence rate ratios* |  |  |  |  |  |  |  |  |
| Unadjusted | 1.0 (Ref.) | 0.99 (0.81-1.20) | 0.92 (0.75-1.13) | 0.32 | 1.0 (Ref.) | 0.97 (0.83-1.12) | 0.90 (0.77-1.05) | 0.17 |
| Fully- Adjusted | 1.0 (Ref.) | 0.95 (0.76-1.20) | 0.85 (0.67-1.08) | 0.22 | 1.0 (Ref.) | 0.89 (0.75-1.06) | 0.86 (0.72-1.03) | 0.19 |

*All models stratified by clinic cluster.

Adjustment for age at enrollment, gender, race, comorbidity score (ICED), obesity status, diabetes, CVD, residual kidney function (self-reported ability to produce >1 cup of urine daily), albumin, creatinine, phosphate.

Table ST2: Crude estimates and incidence rate ratios of all **sepsis hospitalizations** for free and total p-Cresol sulfate stratified by GI disease in HEMO

|  | **Free** | | | | **Total** | | | | | | |
| --- | --- | --- | --- | --- | --- | --- | --- | --- | --- | --- | --- |
|  | **GI Disease** | | | | | | | | | | |
|  | **Lowest**  **(0.00-0.16 mg/dl)** | **Middle**  **(0.16-0.29 mg/dl)** | **Highest**  **(0.29-1.09 mg/dl)** | **PTrend** | **Lowest**  **(0.13-2.30 mg/dl)** | | **Middle**  **(2.30-3.45 mg/dl)** | | **Highest**  **(3.45-6.33 mg/dl)** | | **PTrend** |
| No. of persons | 25 | 25 | 25 |  | 25 | | 25 | | 25 | |  |
| No. of infectious admissions | 35 | 36 | 33 |  | 36 | | 31 | | 37 | |  |
| Total person-years | 122.55 | 121.82 | 123.11 |  | 126.65 | | 115.20 | | 125.63 | |  |
| Incidence rate (per 1000 person-years) | 285.59 | 295.51 | 268.05 |  | 240.84 | | 269.09 | | 294.51 | |  |
| Incidence rate ratios* |  |  |  |  |  | |  | |  | |  |
| Unadjusted | Ref. | 0.89 (0.57-1.38) | 1.18 (0.79-1.76) | 0.40 | Ref. | | 1.01 (0.64-1.57) | | 1.12 (0.74-1.69) | | 0.59 |
| Fully- Adjusted | Ref. | 1.17 (0.66-2.08) | 1.52 (0.90-2.57) | 0.12 | Ref. | | 1.05 (0.58-1.90) | | 1.34 (0.78-2.30) | | 0.28 |
|  |  |  |  |  |  | |  | |  | |  |
|  | **No-GI Disease** | | | | | | | | | | |
|  | **Lowest**  **(0.04-0.18 mg/dl)** | **Middle**  **(0.18-0.30 mg/dl)** | **Highest**  **(0.30-1.02 mg/dl)** |  | **Lowest**  **(0.50-2.68 mg/dl)** | **Middle**  **(2.68-3.95 mg/dl)** | | **Highest**  **(3.95-9.34 mg/dl)** | |  | |
| No. of persons | 45 | 44 | 44 |  | 45 | 44 | | 44 | |  | |
| No. of infectious admissions | 80 | 68 | 66 |  | 77 | 76 | | 61 | |  | |
| Total person-years | 225.15 | 213.65 | 220.29 |  | 230.25 | 217.99 | | 210.85 | |  | |
| Incidence rate (per 1000 person-years) | 355.31 | 318.28 | 299.61 |  | 334.42 | 348.64 | | 289.30 | |  | |
| Incidence rate ratios* |  |  |  |  |  |  | |  | |  | |
| Unadjusted | Ref. | 0.67 (0.51-0.89) | 0.76 (0.58-1.01) | 0.04 | Ref. | 1.02 (0.79-1.31) | | 0.89 (0.69-1.15) | | 0.38 | |
| Fully- Adjusted | Ref. | 0.66 (0.47-0.93) | 0.77 (0.56-1.07) | 0.03 | Ref. | 1.01 (0.77-1.33) | | 0.93 (0.71-1.22) | | 0.59 | |

*All models stratified by clinic cluster.

Adjustment for age at enrollment, gender, race, comorbidity score (ICED), obesity status, diabetes, CVD, residual kidney function (self-reported ability to produce >1 cup of urine daily), albumin, creatinine, phosphate.

Table ST 3: Crude estimates and incidence rate ratios of all participants with **infectious hospitalizations** for free and total P-cresol sulfate stratified by GI disease in CHOICE

|  | **Free** | | | | **Total** | | | |
| --- | --- | --- | --- | --- | --- | --- | --- | --- |
|  | **GI Disease** | | | | | | | |
|  | **Lowest**  **(0.0-0.13 mg/dl)** | **Middle**  **(0.13-0.31 mg/dl)** | **Highest**  **(0.31-3.64 mg/dl)** | **PTrend** | **Lowest**  **(0.0-2.16 mg/dl)** | **Middle**  **(2.16-3.69 mg/dl)** | **Highest**  **(3.69-8.29 mg/dl)** | **PTrend** |
| No. of persons | 73 | 73 | 72 |  | 73 | 73 | 72 |  |
| No. of infectious admissions | 102 | 96 | 128 |  | 89 | 134 | 103 |  |
| Total person-years | 248.51 | 226.49 | 189.86 |  | 227.90 | 228.38 | 208.58 |  |
| Incidence rate (per 1000 person-years) | 410.44 | 423.87 | 674.17 |  | 390.52 | 586.75 | 493.81 |  |
| Incidence rate ratios* |  |  |  |  |  |  |  |  |
| Unadjusted | 1.0 (Ref.) | 1.29 (0.98-1.70) | 0.97 (0.72-1.30) | 0.11 | 1.0 (Ref.) | 1.15 (0.85-1.55) | 1.35 (1.03-1.80) | 0.02 |
| Fully- Adjusted | 1.0 (Ref.) | 1.35 (0.88-2.05) | 1.04 (0.71-1.53) | 0.18 | 1.0 (Ref.) | 1.28 (0.82-1.99) | 1.45 (0.77-2.73) | 0.09 |
|  |  |  |  |  |  |  |  |  |
|  | **No-GI Disease** | | | | | | | |
|  | **Lowest**  **(0.0-0.15 mg/dl)** | **Middle**  **(0.15-0.32 mg/dl)** | **Highest**  **(0.32-2.58 mg/dl)** |  | **Lowest**  **(0.0-2.36 mg/dl)** | **Middle**  **(2.36-4.03 mg/dl)** | **Highest**  **(4.03-11.12 mg/dl)** |  |
| No. of persons | 101 | 101 | 101 |  | 101 | 101 | 101 |  |
| No. of infectious admissions | 123 | 105 | 151 |  | 112 | 118 | 149 |  |
| Total person-years | 396.22 | 305.51 | 365.63 |  | 369.18 | 326.93 | 371.26 |  |
| Incidence rate (per 1000 person-years) | 310.44 | 343.69 | 412.98 |  | 303.38 | 360.93 | 401.34 |  |
| Incidence rate ratios* |  |  |  |  |  |  |  |  |
| Unadjusted | 1.0 (Ref.) | 1.21 (0.92-1.60) | 1.32 (1.02-1.71) | 0.02 | 1.0 (Ref.) | 1.24 (0.94-1.62) | 1.41 (1.08-1.83) | 0.07 |
| Fully- Adjusted | 1.0 (Ref.) | 1.20 (0.86-1.67) | 1.22 (0.89-1.67) | 0.05 | 1.0 (Ref.) | 1.28 (0.93-1.77) | 1.49 (1.02-2.17) | 0.14 |

*All models stratified by clinic cluster.

Adjustment for age at enrollment, gender, race, comorbidity score (ICED), obesity status, diabetes, CVD, residual kidney function (self-reported ability to produce >1 cup of urine daily), albumin, creatinine, phosphate.

Extreme values with either PCS or IS above two standard deviation of the mean for external data and percent free PCS or IS values >15% of total concentration were excluded from the analysis.

Table ST 4: Crude estimates and incidence rate ratios of all participants for **sepsis hospitalizations** for free and total P-cresol sulfate stratified by GI disease in CHOICE

|  | **Free** | | | | **Total** | | | |
| --- | --- | --- | --- | --- | --- | --- | --- | --- |
|  | **GI Disease** | | | | | | | |
|  | **Lowest**  **(0.0-0.13 mg/dl)** | **Middle**  **(0.13-0.31 mg/dl)** | **Highest**  **(0.31-3.64 mg/dl)** | **PTrend** | **Lowest**  **(0.0-2.16 mg/dl)** | **Middle**  **(2.16-3.69 mg/dl)** | **Highest**  **(3.69-8.29 mg/dl)** | **PTrend** |
| No. of persons | 73 | 73 | 72 |  | 73 | 73 | 72 |  |
| No. of infectious admissions | 29 | 29 | 40 |  | 17 | 47 | 34 |  |
| Total person-years | 248.51 | 226.49 | 189.86 |  | 227.90 | 228.38 | 208.58 |  |
| Incidence rate (per 1000 person-years) | 116.69 | 128.04 | 210.68 |  | 74.59 | 205.80 | 163.00 |  |
| Incidence rate ratios* |  |  |  |  |  |  |  |  |
| Unadjusted | 1.0 (Ref.) | 1.32 (0.80-2.17) | 0.97 (0.57-1.65) | 0.20 | 1.0 (Ref.) | 1.78 (0.96-3.29) | 2.52 (1.42-4.49) | 0.025 |
| Fully- Adjusted | 1.0 (Ref.) | 1.32 (0.61-2.88) | 1.87 (0.84-4.20) | 0.10 | 1.0 (Ref.) | 1.89 (0.65-5.54) | 1.90 (0.81-4.41) | 0.09 |
|  |  |  |  |  |  |  |  |  |
|  | **No-GI Disease** | | | | | | | |
|  | **Lowest**  **(0.0-0.15 mg/dl)** | **Middle**  **(0.15-0.32 mg/dl)** | **Highest**  **(0.32-2.58 mg/dl)** |  | **Lowest**  **(0.0-2.39 mg/dl)** | **Middle**  **(2.39-4.06 mg/dl)** | **Highest**  **(4.06-11.12 mg/dl)** |  |
| No. of persons | 101 | 101 | 101 |  | 101 | 101 | 101 |  |
| No. of infectious admissions | 20 | 21 | 27 |  | 22 | 17 | 29 |  |
| Total person-years | 396.22 | 305.51 | 365.63 |  | 369.18 | 326.93 | 371.26 |  |
| Incidence rate (per 1000 person-years) | 50.48 | 68.74 | 73.84 |  | 59.59 | 51.99 | 78.11 |  |
| Incidence rate ratios* |  |  |  |  |  |  |  |  |
| Unadjusted | 1.0 (Ref.) | 1.43 (0.75-2.73) | 1.59 (0.87-2.92) | 0.05 | 1.0 (Ref.) | 0.93 (0.48-1.80) | 1.55 (0.86-2.77) | 0.10 |
| Fully- Adjusted | 1.0 (Ref.) | 1.45 (0.70-2.99) | 1.17 (0.56-2.43) | 0.06 | 1.0 (Ref.) | 0.85 (0.41-1.78) | 1.22 (0.56-2.65) | 0.08 |

*All models stratified by clinic cluster.

Adjustment for age at enrollment, gender, race, comorbidity score (ICED), obesity status, diabetes, CVD, residual kidney function (self-reported ability to produce >1 cup of urine daily), albumin, creatinine, phosphate.

Extreme values with either PCS or IS above two standard deviation of the mean for external data and percent free PCS or IS values >15% of total concentration were excluded from the analysis.

Table ST 5: Crude estimates and incidence rate ratios of all participants with **infectious hospitalizations** for free and total P-cresol sulfate stratified by GI disease in HEMO

|  | **Free** | | | | **Total** | | | | |
| --- | --- | --- | --- | --- | --- | --- | --- | --- | --- |
|  | **GI Disease** | | | | | | | | |
|  | **Lowest**  **(0.00-0.19 mg/dl)** | **Middle**  **(0.19-0.34 mg/dl)** | **Highest**  **(0.34-1.57 mg/dl)** | **PTrend** | **Lowest**  **(0.0-2.48 mg/dl)** | | **Middle**  **(2.48-3.65 mg/dl)** | **Highest**  **(3.65-7.60 mg/dl)** | **PTrend** |
| No. of persons | 65 | 66 | 64 |  | 65 | | 65 | 65 |  |
| No. of infectious admissions | 129 | 129 | 142 |  | 126 | | 147 | 123 |  |
| Total person-years | 295.38 | 314.29 | 273.76 |  | 307.32 | | 286.66 | 293.34 |  |
| Incidence rate (per 1000 person-years) | 436.73 | 410.45 | 518.71 |  | 409.99 | | 512.81 | 419.31 |  |
| Incidence rate ratios* |  |  |  |  |  | |  |  |  |
| Unadjusted | 1.0 (Ref.) | 0.93 (0.68-1.27) | 0.95 (0.70-1.27) | 0.70 | 1.0 (Ref.) | | 0.96 (0.71-1.31) | 1.01 (0.75-1.36) | 0.51 |
| Fully- Adjusted | 1.0 (Ref.) | 0.90 (0.61-1.32) | 0.92 (0.63-1.34) | 0.64 | 1.0 (Ref.) | | 0.97 (0.65-1.45) | 1.00 (0.71-1.52) | 0.66 |
|  |  |  |  |  |  | |  |  |  |
|  | **No-GI Disease** | | | | | | | | |
|  | **Lowest**  **(0.0-0.18 mg/dl)** | **Middle**  **(0.18-0.34 mg/dl)** | **Highest**  **(0.34-1.21 mg/dl)** |  | **Lowest**  **(0.0-2.47 mg/dl)** | **Middle**  **(2.47-3.73 mg/dl)** | | **Highest**  **(3.73-9.34 mg/dl)** |  |
| No. of persons | 99 | 97 | 94 |  | 163 | 163 | | 162 |  |
| No. of infectious admissions | 213 | 215 | 183 |  | 362 | 345 | | 306 |  |
| Total person-years | 489.95 | 476.44 | 439.75 |  | 793.67 | 764.05 | | 750.13 |  |
| Incidence rate (per 1000 person-years) | 434.74 | 451.26 | 416.14 |  | 456.11 | 451.54 | | 407.93 |  |
| Incidence rate ratios* |  |  |  |  |  |  | |  |  |
| Unadjusted | 1.0 (Ref.) | 0.87 (0.68-1.17) | 0.80 (0.62-1.03) | 0.07 | 1.0 (Ref.) | 0.91 (0.71-1.17) | | 0.83 (0.65-1.07) | 0.15 |
| Fully- Adjusted | 1.0 (Ref.) | 0.79 (0.59-1.06) | 0.74 (0.54-1.01) | 0.05 | 1.0 (Ref.) | 0.94 (0.70-1.27) | | 0.95 (0.70-1.28) | 0.72 |

*All models stratified by clinic cluster.

Adjustment for age at enrollment, gender, race, comorbidity score (ICED), obesity status, diabetes, CVD, residual kidney function (self-reported ability to produce >1 cup of urine daily), albumin, creatinine, phosphate.

Extreme values with either PCS or IS above two standard deviation of the mean for external data and percent free PCS or IS values >15% of total concentration were excluded from the analysis.

Table ST 6: Crude estimates and incidence rate ratios of all participants for **sepsis hospitalizations** for free and total P-cresol sulfate stratified by GI disease in HEMO

|  | **Free** | | | | **Total** | | | | | | |
| --- | --- | --- | --- | --- | --- | --- | --- | --- | --- | --- | --- |
|  | **GI Disease** | | | | | | | | | | |
|  | **Lowest**  **(0.01-0.19 mg/dl)** | **Middle**  **(0.19-0.32 mg/dl)** | **Highest**  **(0.32-1.57 mg/dl)** | **PTrend** | **Lowest**  **(0.0-2.31 mg/dl)** | | **Middle**  **(2.31-3.46 mg/dl)** | | **Highest**  **(3.46-6.95 mg/dl)** | | **PTrend** |
| No. of persons | 36 | 34 | 35 |  | 35 | | 35 | | 35 | |  |
| No. of infectious admissions | 53 | 49 | 57 |  | 54 | | 48 | | 56 | |  |
| Total person-years | 171.21 | 169.49 | 157.18 |  | 176.09 | | 156.11 | | 168.13 | |  |
| Incidence rate (per 1000 person-years) | 309.55 | 289.11 | 362.65 |  | 306.66 | | 307.47 | | 333.07 | |  |
| Incidence rate ratios* |  |  |  |  |  | |  | |  | |  |
| Unadjusted | 1.0 (Ref.) | 1.07 (0.63-1.79) | 0.93 (0.55-1.55) | 0.78 | 1.0 (Ref.) | | 0.97 (0.55-1.73) | | 1.03 (0.62-1.72) | | 0.90 |
| Fully- Adjusted | 1.0 (Ref.) | 1.12 (0.53-2.39) | 1.34 (0.63-2.87) | 0.45 | 1.0 (Ref.) | | 1.14 (0.53-2.45) | | 1.46 (0.67-3.18) | | 0.33 |
|  |  |  |  |  |  | |  | |  | |  |
|  | **No-GI Disease** | | | | | | | | | | |
|  | **Lowest**  **(0.0-0.20 mg/dl)** | **Middle**  **(0.21-0.35 mg/dl)** | **Highest**  **(0.35-1.13 mg/dl)** |  | **Lowest**  **(0.01-2.54 mg/dl)** | **Middle**  **(2.54-3.95 mg/dl)** | | **Highest**  **(3.95-9.34 mg/dl)** | |  | |
| No. of persons | 66 | 65 | 65 |  | 66 | 66 | | 65 | |  | |
| No. of infectious admissions | 129 | 89 | 99 |  | 111 | 115 | | 92 | |  | |
| Total person-years | 328.28 | 327.88 | 313.50 |  | 328.70 | 330.62 | | 314.16 | |  | |
| Incidence rate (per 1000 person-years) | 392.96 | 271.44 | 315.79 |  | 337.70 | 347.83 | | 292.84 | |  | |
| Incidence rate ratios* |  |  |  |  |  |  | |  | |  | |
| Unadjusted | 1.0 (Ref.) | 0.84 (0.59-1.18) | 0.82 (0.58-1.16) | 0.25 | 1.0 (Ref.) | 1.05 (0.74-1.48) | | 0.80 (0.57-1.14) | | 0.22 | |
| Fully- Adjusted | 1.0 (Ref.) | 1.07 (0.66-1.74) | 0.95 (0.61-1.47) | 0.76 | 1.0 (Ref.) | 1.30 (0.84-2.01) | | 1.05 (0.67-2.67) | | 0.82 | |

*All models stratified by clinic cluster.

Adjustment for age at enrollment, gender, race, comorbidity score (ICED), obesity status, diabetes, CVD, residual kidney function (self-reported ability to produce >1 cup of urine daily), albumin, creatinine, phosphate.

Extreme values with either PCS or IS above two standard deviation of the mean for external data and percent free PCS or IS values >15% of total concentration were excluded from the analysis.

| Characteristic | Free Indoxyl Sulfate (0.0-0.59 mg/dL) | | | | Total Indoxyl Sulfate (0.06-6.82 mg/dL) | | | |
| --- | --- | --- | --- | --- | --- | --- | --- | --- |
|  | Lowest  (0.0-0.07) | Middle (0.07-0.14) | Highest  (0.14-0.59) | P Value | Lowest  (0.06-1.20) | Middle (1.20-1.96) | Highest  (1.96-6.82) | P Value |
| N (%) | 132 (33.5) | 131 (33.3) | 131 (33.3) |  | 132 (33.5) | 131 (33.3) | 131 (33.3) |  |
| Age, mean (SD) | 55.5 (14.5) | 56.9 (14.6) | 59.1 (15.6) | 0.13 | 57.4 (14.3) | 55.6 (15.2) | 58.4 (15.3) | 0.31 |
| Race, % |  |  |  | 0.13 |  |  |  | 0.49 |
| White | 56.8 | 56.5 | 64.9 |  | 56.8 | 57.3 | 64.1 |  |
| African American | 40.2 | 38.2 | 27.5 |  | 39.4 | 35.9 | 30.5 |  |
| Male, % | 57.6 | 52.7 | 54.2 | 0.72 | 53.8 | 53.4 | 57.3 | 0.79 |
| Severe comorbidity score, % | 25.8 | 31.3 | 22.9 | 0.27 | 28.8 | 30.5 | 20.6 | 0.23 |
| BMI, mean(SD), kg/m2 | 27.8 (7.4) | 27.8 (6.7) | 27.0 (6.4) | 0.60 | 28.1 (7.2) | 27.7 (7.3) | 26.8 (5.9) | 0.31 |
| <18, % | 3.9 | 0.8 | 2.5 |  | 4.8 | 1.6 | 0.8 |  |
| 18-25, % | 38.6 | 39.5 | 42.2 |  | 32.8 | 41.6 | 45.9 |  |
| >=25, % | 57.5 | 59.7 | 55.4 |  | 62.4 | 56.8 | 53.3 |  |
| Blood Pressure, % |  |  |  | 0.57 |  |  |  | 0.52 |
| SBP<130 | 7.6 | 9.4 | 10.9 |  | 7.6 | 9.3 | 10.9 |  |
| 130<=SBP<160 | 64.4 | 58.6 | 54.3 |  | 64.9 | 58.1 | 54.3 |  |
| SBP>=160 | 28.0 | 32.0 | 34.9 |  | 27.5 | 32.6 | 34.9 |  |
| Kt/V, median (Q1-Q3) | 1.5 (1.3-1.6) | 1.5 (1.3-1.6) | 1.5 (0.2) | 0.41 | 1.5 (1.3-1.6) | 1.4 (1.2-1.5) | 1.5 (1.4-1.6) | 0.02 |
| Diabetes, % | 53.0 | 61.1 | 45.8 | 0.04 | 59.1 | 60.3 | 40.5 | 0.001 |
| Gastrointestinal Disease, % | 40.9 | 43.5 | 38.9 | 0.75 | 42.4 | 41.2 | 39.7 | 0.90 |
| RKF (%UO> 1 cup at bl) | 87.4 | 84.3 | 77.8 | 0.11 | 89.1 | 79.8 | 80.5 | 0.09 |
| Laboratory |  |  |  |  |  |  |  |  |
| Albumin, median (Q1-Q3), g/dl | 3.7 (3.5-3.9) | 3.7 (3.4-3.9) | 3.9 (3.6-4.0) | 0.24 | 3.7 (3.6-3.9) | 3.6 (3.4-3.9) | 3.9 (3.6-4.0) | 0.25 |
| Creatinine, mean (SD), mg/dl | 7.4 (2.6) | 8.9 (2.9) | 8.8 (2.2) | 0.01 | 7.3 (2.5) | 8.5 (2.5) | 9.2 (2.7) | 0.001 |
| Phosphorus, mean (SD), mg/dl | 5.0 (1.3) | 5.4 (1.3) | 5.2 (1.7) | 0.47 | 4.9 (1.2) | 5.5 (1.7) | 5.2 (1.4) | 0.25 |
| C-reactive protein, median (Q1-Q3),  mg/dl | 0.4 (0.2-1.0) | 0.4 (0.2-0.9) | 0.4 (0.1-0.7) | 0.89 | 0.4 (0.2-1.1) | 0.4 (0.2-0.9) | 0.3 (0.1-0.6) | 0.26 |
| Calcium, mean (SD), mg/dl | 9.1 (0.9) | 9.0 (0.8) | 9.2 (0.8) | 0.56 | 9.3 (0.8) | 8.9 (0.9) | 9.1 (0.8) | 0.15 |
| Total Cholesterol, mean (SD), mg/dl | 173.9 (40.5) | 180.3 (49.6) | 175.2 (26.4) | 0.88 | 176.6 (45.2) | 163.9 (35.6) | 184.5 (30.5) | 0.27 |
| Triglycerides, mean (SD), mg/dl | 191.7 (170.9) | 168.4 (115.8) | 244.6 (215.2) | 0.41 | 201.4 (172.9) | 225.7 (245.4) | 196.9 (127.4) | 0.88 |
| Vascular Access Type, % |  |  |  | 0.95 |  |  |  | 0.38 |
| Arteriovenous Fistula | 17.0 | 15.9 | 16.1 |  | 16.1 | 11.5 | 21.4 |  |
| Arteriovenous Graft | 23.2 | 24.8 | 20.5 |  | 24.1 | 23.9 | 20.5 |  |
| Central Venous Catheter | 59.8 | 29.3 | 63.4 |  | 59.8 | 64.6 | 58.1 |  |
| Numbers are presented as mean (SD) or number (%) unless otherwise indicated. P values by ANOVA in case continuous variables are normally distributed and Kruskal-Wallis test in case continuous variables are not normally distributed and χ2 tests for categorical variables. Fisher’s exact test was used when expected cell frequency was less than 5. | | | | | | | | |

Table ST 7: Selected Characteristics of study participants by levels of free and total Indoxyl Sulfate in the Choices for Healthy Outcomes in Caring for ESRD Study (N=394)

Table ST 8: Selected Characteristics of study participants by levels free and total Indoxyl Sulfate in the HEMO Study (N=347)

| Characteristic | Free Indoxyl Sulfate (0.01-0.63 mg/dL) | | | | Total Indoxyl Sulfate (0.19-11.56 mg/dL) | | | |
| --- | --- | --- | --- | --- | --- | --- | --- | --- |
|  | Lowest  (0.01-0.19) | Middle  (0.19-0.34) | Highest (0.34-0.63) | P Value | Lowest (0.19-2.26) | Middle  (2.26-3.48) | Highest (3.48-11.56) | P Value |
| N (%) | 116 (33.4) | 116 (33.4) | 115 (33.1) |  | 116 (33.4) | 116 (33.4) | 115 (33.1) |  |
| Age, mean (SD) | 59.1 (13.6) | 59.6 (13.0) | 57.9 (15.8) | 0.67 | 60.7 (12.4) | 59.4 (14.3) | 56.6 (15.5) | 0.07 |
| Males, % | 41.4 | 37.1 | 50.4 | 0.11 | 40.5 | 37.9 | 50.4 | 0.12 |
| African Americans, % | 62.1 | 65.5 | 65.2 | 0.83 | 60.3 | 68.9 | 63.5 | 0.38 |
| Body Mass Index, % |  |  |  | 0.91 |  |  |  | 0.60 |
| <18 | 48.3 | 46.6 | 43.5 |  | 50.0 | 43.9 | 44.3 |  |
| 18-25 | 49.1 | 49.1 | 53.0 |  | 48.3 | 50.9 | 52.2 |  |
| >=25 | 2.6 | 4.3 | 3.5 |  | 1.7 | 5.2 | 3.5 |  |
| ICED, % |  |  |  | 0.45 |  |  |  | 0.14 |
| 0-1 | 39.7 | 37.9 | 30.4 |  | 43.1 | 34.5 | 30.4 |  |
| 2 | 28.5 | 31.0 | 28.7 |  | 29.3 | 31.9 | 27.0 |  |
| 3 | 31.9 | 31.1 | 40.9 |  | 27.6 | 33.6 | 42.6 |  |
| Blood Pressure, % |  |  |  | 0.97 |  |  |  | 0.98 |
| SBP<130 | 35.3 | 36.2 | 33.9 |  | 35.3 | 35.3 | 34.8 |  |
| 130<=SBP<160 | 52.6 | 52.6 | 52.2 |  | 50.9 | 52.6 | 53.9 |  |
| SBP>=160 | 12.1 | 11.2 | 13.9 |  | 13.8 | 12.1 | 11.3 |  |
| Laboratory |  |  |  |  |  |  |  |  |
| Albumin, median (Q1-Q3), g/dl | 3.9 (3.6-4.1) | 3.9 (3.7-4.1) | 3.9 (3.6-4.1) | 0.88 | 3.8 (3.6-4.0) | 3.9 (3.7-4.1) | 3.9 (3.7-4.1) | 0.02 |
| Creatinine, mean (SD),mg/dL | 9.6 (3.4) | 10.3 (2.5) | 11.3 (2.8) | 0.002 | 9.2 (2.9) | 10.3 (2.7) | 11.7 (2.7) | <0.0001 |
| Phosphorus, mean (SD), mg/dl | 5.5 (1.6) | 5.7 (1.9) | 5.9 (1.8) | 0.25 | 5.3 (1.6) | 5.7 (1.8) | 5.9 (1.9) | 0.02 |
| Calcium, mean (SD), mg/dl | 9.2 (1.0) | 9.3 (1.1) | 9.6 (0.8) | 0.002 | 9.2 (1.0) | 9.2 (0.9) | 9.7 (0.9) | 0.001 |
| Total Cholesterol, mean(SD), mg/dl | 166.2 (38.1) | 172.0 (37.7) | 170.7 (40.7) | 0.55 | 167.4 (36.4) | 174.3 (40.4) | 167.3 (39.4) | 0.38 |
| Relative Volume Removed, mean (SD) | 4.2 (1.9) | 3.9 (1.7) | 4.2 (1.6) | 0.38 | 3.9 (1.7) | 4.4 (1.9) | 4.1 (1.7) | 0.12 |
| Residual Kidney Function (urea clearance in ml/min), mean (SD) | 0.4 (0.5) | 0.3 (0.5) | 0.2 (0.4) | 0.07 | 0.4 (0.5) | 0.3 (0.5) | 0.2 (0.4) | 0.03 |
| Vascular Access Type, % |  |  |  | 0.22 |  |  |  | 0.18 |
| Arteriovenous Fistula | 30.1 | 28.8 | 33.9 |  | 26.6 | 27.9 | 38.5 |  |
| Arteriovenous Graft | 60.2 | 68.5 | 59.6 |  | 64.6 | 67.6 | 56.0 |  |
| Central Venous Catheter | 9.7 | 2.7 | 6.4 |  | 8.9 | 4.5 | 5.5 |  |
| Numbers are presented as mean (SD) or number (%) unless otherwise indicated. P values by ANOVA in case continuous variables are normally distributed and Kruskal-Wallis test in case continuous variables are not normally distributed and χ2 tests for categorical variables. Fisher’s exact test was used when expected cell frequency was less than 5. | | | | | | | | |

Table ST 9: Crude estimates and incidence rate ratios of all **infectious hospitalizations** for free and total indoxyl sulfate stratified by GI disease in CHOICE

|  | **Free** | | | | **Total** | | | |
| --- | --- | --- | --- | --- | --- | --- | --- | --- |
|  | **GI Disease** | | | | | | | |
|  | **Lowest**  **(0.0-0.07 mg/dl)** | **Middle**  **(0.07-0.14 mg/dl)** | **Highest**  **(0.14-0.48 mg/dl)** | **PTrend** | **Lowest**  **(0.06-1.20 mg/dl)** | **Middle**  **(1.20-1.89 mg/dl)** | **Highest**  **(1.89-6.82 mg/dl)** | **PTrend** |
| No. of persons | 54 | 54 | 54 |  | 54 | 54 | 54 |  |
| No. of infectious admissions | 75 | 87 | 79 |  | 58 | 111 | 72 |  |
| Total person-years | 185.47 | 168.75 | 175.24 |  | 165.68 | 189.34 | 174.45 |  |
| Incidence rate (per 1000 person-years) | 404.37 | 515.56 | 450.80 |  | 350.08 | 586.25 | 412.72 |  |
| Incidence rate ratios* |  |  |  |  |  |  |  |  |
| Unadjusted | 1.0 (Ref.) | 1.27 (0.99-1.67) | 1.11 (0.84-1.55) | 0.10 | 1.0 (Ref.) | 1.67 (1.18-2.08) | 1.17 (0.72-1.61) | 0.17 |
| Fully- Adjusted | 1.0 (Ref.) | 1.02 (0.57-1.82) | 0.77 (0.34-1.77) | 0.53 | 1.0 (Ref.) | 1.11 (0.64-2.17) | 0.86 (0.51-1.52) | 0.35 |
|  |  |  |  |  |  |  |  |  |
|  | **No-GI Disease** | | | | | | | |
|  | **Lowest**  **(0.01-0.07 mg/dl)** | **Middle**  **(0.07-0.14 mg/dl)** | **Highest**  **(0.14-0.60 mg/dl)** |  | **Lowest**  **(0.12-1.23 mg/dl)** | **Middle**  **(1.23-2.02 mg/dl)** | **Highest**  **(2.02-5.97 mg/dl)** |  |
| No. of persons | 78 | 77 | 77 |  | 78 | 77 | 77 |  |
| No. of infectious admissions | 96 | 102 | 72 |  | 98 | 100 | 72 |  |
| Total person-years | 289.45 | 285.02 | 249.50 |  | 283.96 | 282.61 | 257.40 |  |
| Incidence rate (per 1000 person-years) | 331.67 | 357.87 | 288.57 |  | 345.12 | 353.85 | 279.72 |  |
| Incidence rate ratios* |  |  |  |  |  |  |  |  |
| Unadjusted | 1.0 (Ref.) | 1.17 (0.87-1.57) | 0.89 (0.63-1.57) | 0.59 | 1.0 (Ref.) | 1.12 (0.83-1.52) | 0.85 (0.60-1.19) | 0.37 |
| Fully- Adjusted | 1.0 (Ref.) | 1.22 (0.73-2.04) | 0.99 (0.50-1.93) | 0.99 | 1.0 (Ref.) | 1.04 (0.66-1.64) | 1.09 (0.63-1.90) | 0.15 |

*All models stratified by clinic cluster.

Adjustment for age at enrollment, gender, race, comorbidity score (ICED), obesity status, diabetes, CVD, residual kidney function (self-reported ability to produce >1 cup of urine daily), albumin, creatinine, phosphate.

Table ST 10: Crude estimates and incidence rate ratios of all **sepsis hospitalizations** for free and total indoxyl sulfate stratified by GI disease in CHOICE

|  | **Free** | | | | **Total** | | | |
| --- | --- | --- | --- | --- | --- | --- | --- | --- |
|  | **GI Disease** | | | | | | | |
|  | **Lowest**  **(0.0-0.07 mg/dl)** | **Middle**  **(0.07-0.14 mg/dl)** | **Highest**  **(0.14-0.48 mg/dl)** | **PTrend** | **Lowest**  **(0.06-1.20 mg/dl)** | **Middle**  **(1.20-1.89 mg/dl)** | **Highest**  **(1.89-6.82 mg/dl)** | **PTrend** |
| No. of persons | 54 | 54 | 54 |  | 54 | 54 | 54 |  |
| No. of infectious admissions | 12 | 30 | 35 |  | 14 | 28 | 35 |  |
| Total person-years | 185.47 | 168.75 | 175.24 |  | 165.68 | 189.34 | 174.45 |  |
| Incidence rate (per 1000 person-years) | 64.69 | 177.78 | 199.72 |  | 84.50 | 147.88 | 200.63 |  |
| Incidence rate ratios* |  |  |  |  |  |  |  |  |
| Unadjusted | 1.0 (Ref.) | 1.55 (0.75-3.20) | 1.84 (0.90-3.76) | 0.41 | 1.0 (Ref.) | 1.07 (0.53-2.16) | 1.32 (0.67-2.59) | 0.35 |
| Fully- Adjusted | 1.0 (Ref.) | 1.62 (0.44-3.88) | 2.33 (0.84-4.48) | 0.26 | 1.0 (Ref.) | 0.96 (0.35-2.66) | 0.88 (0.27-2.82) | 0.84 |
|  |  |  |  |  |  |  |  |  |
|  | **No-GI Disease** | | | | | | | |
|  | **Lowest**  **(0.01-0.07 mg/dl)** | **Middle**  **(0.07-0.14 mg/dl)** | **Highest**  **(0.14-0.60 mg/dl)** |  | **Lowest**  **(012-1.23 mg/dl)** | **Middle**  **(1.23-2.02 mg/dl)** | **Highest**  **(2.02-5.97 mg/dl)** |  |
| No. of persons | 78 | 77 | 77 |  | 78 | 77 | 77 |  |
| No. of infectious admissions | 13 | 18 | 21 |  | 11 | 22 | 19 |  |
| Total person-years | 289.45 | 285.02 | 249.50 |  | 283.96 | 282.61 | 257.40 |  |
| Incidence rate (per 1000 person-years) | 44.91 | 63.15 | 84.17 |  | 38.74 | 77.84 | 73.81 |  |
| Incidence rate ratios* |  |  |  |  |  |  |  |  |
| Unadjusted | 1.0 (Ref.) | 1.57 (0.75-3.30) | 2.09 (0.97-4.49) | 0.06 | 1.0 (Ref.) | 2.39 (1.11-5.17) | 1.95 (0.87-4.37) | 0.13 |
| Fully- Adjusted | 1.0 (Ref.) | 1.17 (0.42-3.23) | 1.50 (0.46-4.92) | 0.49 | 1.0 (Ref.) | 1.54 (0.54-4.38) | 1.76 (0.56-4.46) | 0.34 |

*All models stratified by clinic cluster.

Adjustment for age at enrollment, gender, race, comorbidity score (ICED), obesity status, diabetes, CVD, residual kidney function (self-reported ability to produce >1 cup of urine daily), albumin, creatinine, phosphate.

|  | **Free** | | | | **Total** | | | |
| --- | --- | --- | --- | --- | --- | --- | --- | --- |
|  | **GI Disease** | | | | | | | |
|  | **Lowest**  **(0.01-0.18 mg/dl)** | **Middle**  **(0.18-0.33 mg/dl)** | **Highest**  **(0.33-0.62 mg/dl)** | **PTrend** | **Lowest**  **(0.19-2.09 mg/dl)** | **Middle**  **(2.09-3.29 mg/dl)** | **Highest**  **(3.29-6.00 mg/dl)** | **PTrend** |
| No. of persons | 48 | 47 | 47 |  | 48 | 47 | 47 |  |
| No. of infectious admissions | 100 | 80 | 92 |  | 96 | 90 | 86 |  |
| Total person-years | 205.75 | 220.39 | 237.24 |  | 211.78 | 207.64 | 243.96 |  |
| Incidence rate (per 1000 person-years) | 486.03 | 362.99 | 387.79 |  | 453.31 | 433.45 | 352.51 |  |
| Incidence rate ratios* |  |  |  |  |  |  |  |  |
| Unadjusted | 1.0 (Ref.) | 0.79 (0.58-1.09) | 0.82 (0.62-1.10) | 0.19 | 1.0 (Ref.) | 0.89 (0.66-1.22) | 0.82 (0.61-1.11) | 0.20 |
| Fully- Adjusted | 1.0 (Ref.) | 0.74 (0.51-1.08) | 0.82 (0.57-1.18) | 0.31 | 1.0 (Ref.) | 0.85 (0.57-1.26) | 0.87 (0.58-1.30) | 0.51 |
|  |  |  |  |  |  |  |  |  |
|  | **No-GI Disease** | | | | | | | |
|  | **Lowest**  **(0.04-0.21 mg/dl)** | **Middle**  **(0.21-0.35 mg/dl)** | **Highest**  **(0.35-0.63 mg/dl)** |  | **Lowest**  **(0.63-2.34 mg/dl)** | **Middle**  **(2.34-3.67 mg/dl)** | **Highest**  **(3.67-11.57 mg/dl)** |  |
| No. of persons | 69 | 68 | 68 |  | 69 | 68 | 68 |  |
| No. of infectious admissions | 146 | 146 | 117 |  | 140 | 160 | 109 |  |
| Total person-years | 332.92 | 337.98 | 335.36 |  | 326.63 | 344.34 | 335.29 |  |
| Incidence rate (per 1000 person-years) | 438.54 | 431.98 | 348.88 |  | 428.62 | 464.66 | 325.09 |  |
| Incidence rate ratios* |  |  |  |  |  |  |  |  |
| Unadjusted | 1.0 (Ref.) | 0.99 (0.77-1.27) | 0.77 (0.60-0.99) | 0.05 | 1.0 (Ref.) | 1.10 (0.87-1.40) | 0.75 (0.58-0.97) | 0.03 |
| Fully- Adjusted | 1.0 (Ref.) | 1.10 (0.84-1.45) | 0.82 (0.61-1.11) | 0.26 | 1.0 (Ref.) | 1.27 (0.96-1.67) | 0.84 (0.61-1.16) | 0.36 |

Table ST11: Crude estimates and incidence rate ratios of all **infectious hospitalizations** for free and total Indoxyl sulfate stratified by GI disease in HEMO

*All models stratified by clinic cluster.

Adjustment for age at enrollment, gender, race, comorbidity score (ICED), obesity status, diabetes, CVD, residual kidney function (self-reported ability to produce >1 cup of urine daily), albumin, creatinine, phosphate.

Table ST12: Crude estimates and incidence rate ratios of all **sepsis hospitalizations** for free and total Indoxyl sulfate stratified by GI disease in HEMO

|  | **Free** | | | | **Total** | | | | | | |
| --- | --- | --- | --- | --- | --- | --- | --- | --- | --- | --- | --- |
|  | **GI Disease** | | | | | | | | | | |
|  | **Lowest**  **(0.01-0.18 mg/dl)** | **Middle**  **(0.18-0.33 mg/dl)** | **Highest**  **(0.33-0.62 mg/dl)** | **PTrend** | **Lowest**  **(0.20-2.08 mg/dl)** | | **Middle**  **(2.08-3.36 mg/dl)** | | **Highest**  **(3.36-5.65 mg/dl)** | | **PTrend** |
| No. of persons | 25 | 25 | 25 |  | 25 | | 25 | | 25 | |  |
| No. of infectious admissions | 37 | 33 | 34 |  | 39 | | 32 | | 33 | |  |
| Total person-years | 112.04 | 122.51 | 132.94 |  | 115.95 | | 114.07 | | 137.47 | |  |
| Incidence rate (per 1000 person-years) | 330.23 | 269.37 | 255.76 |  | 336.36 | | 280.52 | | 240.06 | |  |
| Incidence rate ratios* |  |  |  |  |  | |  | |  | |  |
| Unadjusted | Ref. | 0.83 (0.48-1.43) | 0.74 (0.45-1.24) | 0.25 | Ref. | | 0.79 (0.48-1.30) | | 0.69 (0.41-1.15) | | 0.14 |
| Fully- Adjusted | Ref. | 1.12 (0.50-2.52) | 1.09 (0.49-2.42) | 0.86 | Ref. | | 1.27 (0.49-3.33) | | 1.05 (0.41-2.69) | | 0.97 |
|  |  |  |  |  |  | |  | |  | |  |
|  | **No-GI Disease** | | | | | | | | | | |
|  | **Lowest**  **(0.04-0.21 mg/dl)** | **Middle**  **(0.21-0.33 mg/dl)** | **Highest**  **(0.33-0.63 mg/dl)** |  | **Lowest**  **(0.63-2.36 mg/dl)** | **Middle**  **(2.36-3.42 mg/dl)** | | **Highest**  **(3.42-11.57 mg/dl)** | |  | |
| No. of persons | 46 | 43 | 44 |  | 45 | 44 | | 44 | |  | |
| No. of infectious admissions | 75 | 69 | 70 |  | 68 | 80 | | 66 | |  | |
| Total person-years | 227.32 | 215.18 | 216.59 |  | 241.70 | 225.47 | | 218.92 | |  | |
| Incidence rate (per 1000 person-years) | 329.93 | 320.66 | 323.20 |  | 316.72 | 354.81 | | 301.48 | |  | |
| Incidence rate ratios* |  |  |  |  |  |  | |  | |  | |
| Unadjusted | Ref. | 1.12 (0.78-1.61) | 0.97 (0.69-1.36) | 0.87 | Ref. | 1.13 (0.81-1.57) | | 0.95 (0.67-1.35) | | 0.77 | |
| Fully- Adjusted | Ref. | 1.46 (0.94-2.27) | 0.93 (0.61-1.44) | 0.76 | Ref. | 1.41 (0.93-2.12) | | 0.97 (0.63-1.50) | | 0.89 | |

*All models stratified by clinic cluster.

Adjustment for age at enrollment, gender, race, comorbidity score (ICED), obesity status, diabetes, CVD, residual kidney function (self-reported ability to produce >1 cup of urine daily), albumin, creatinine, phosphate.
